# MEG-based neurofeedback for hand rehabilitation

**DOI:** 10.1186/s12984-015-0076-7

**Published:** 2015-09-22

**Authors:** Stephen T. Foldes, Douglas J. Weber, Jennifer L. Collinger

**Affiliations:** VA Pittsburgh Healthcare System, Human Engineering Research Laboratories, Pittsburgh, PA 15206 USA; Department of Physical Medicine and Rehabilitation, University of Pittsburgh, Pittsburgh, PA 15213 USA; Center for the Neural Basis of Cognition, Carnegie Mellon University, University of Pittsburgh, Pittsburgh, PA 15213 USA; Department of Bioengineering, University of Pittsburgh, University of Pittsburgh, Pittsburgh, PA 15213 USA

**Keywords:** Brain-Computer Interface, Neurofeedback, Spinal Cord Injury, Magnetoencephalography, Neuroplasticity, Rehabilitation

## Abstract

**Background:**

Providing neurofeedback (NF) of motor-related brain activity in a biologically-relevant and intuitive way could maximize the utility of a brain-computer interface (BCI) for promoting therapeutic plasticity. We present a BCI capable of providing intuitive and direct control of a video-based grasp.

**Methods:**

Utilizing magnetoencephalography’s (MEG) high temporal and spatial resolution, we recorded sensorimotor rhythms (SMR) that were modulated by grasp or rest intentions. SMR modulation controlled the grasp aperture of a stop motion video of a human hand. The displayed hand grasp position was driven incrementally towards a closed or opened state and subjects were required to hold the targeted position for a time that was adjusted to change the task difficulty.

**Results:**

We demonstrated that three individuals with complete hand paralysis due to spinal cord injury (SCI) were able to maintain brain-control of closing and opening a virtual hand with an average of 63 % success which was significantly above the average chance rate of 19 %. This level of performance was achieved without pre-training and less than 4 min of calibration. In addition, successful grasp targets were reached in 1.96 ± 0.15 s. Subjects performed 200 brain-controlled trials in approximately 30 min excluding breaks. Two of the three participants showed a significant improvement in SMR indicating that they had learned to change their brain activity within a single session of NF.

**Conclusions:**

This study demonstrated the utility of a MEG-based BCI system to provide realistic, efficient, and focused NF to individuals with paralysis with the goal of using NF to induce neuroplasticity.

## Background

Typical rehabilitation strategies for people with paralysis rely on residual physical function to drive motor recovery. However, if injury prevents a person from generating muscle activity, an alternative approach is to use a therapy based on extracting information about motor intention directly from the brain. Studies of individuals with spinal cord injury (SCI) or hemiplegia due to stroke have shown that functional recovery during the early stages of rehabilitation is accompanied by a return to a more normal activation pattern within the sensorimotor cortex [1–4]. In the case of SCI, motor-related activity is often altered (see [5] for a summary). Using brain computer interfaces (BCIs), people can learn to modulate their brain activity, which has the potential to promote therapeutic neuroplasticity [6, 7]. BCIs record neural activity and allow for real-time feedback of certain features of the brain signal in order to facilitate learning through the principles of operant conditioning. We expect that improving a person’s ability to generate motor-related brain activity could strengthen any remaining or repaired corticospinal connections, which would better transmit intentions to the paralyzed muscles.

BCIs rely on volitional modulation of cortical activity, often achieved through attempted and imagined movements, and can form the basis of neurofeedback (NF) training paradigms. In particular, sensorimotor rhythms (SMR) (8–30 Hz) have been used to control assistive devices and are being investigated for rehabilitative NF [6, 8, 9]. While most research to date has used electroencephalography (EEG) or electrocorticography (ECoG) to record SMR activity, magnetoencephalography (MEG) can also be used to detect and provide real-time feedback of SMRs [10–15]. MEG has the advantage of non-invasively recording across the whole scalp while maintaining high spatial and temporal resolution. In addition, compared to EEG, MEG allows for better source localization and detection of higher frequencies because magnetic fields are not attenuated by the skull as is the case for electric fields [16]. Though not portable, MEG-based BCIs are relevant for rehabilitation interventions.

BCI-based neurofeedback paradigms have recently been investigated for restoration of motor function after stroke using both EEG [17, 18] and MEG [11] with some success. We present a novel MEG-based BCI system to deliver a NF paradigm for promoting therapeutic neuroplasticity for recovery of hand function. Our system utilizes anthropomorphic feedback to activate intact action-observation networks based on the same principles as mirror therapy for stroke and phantom limb syndrome [19–22]. Another novel feature is that subjects have proportional, continuous control of the real-time display using cortical activity directly associated with the intended movement. The goal was to provide NF using an intuitive and natural control signal based on cortical activity that typically occurs during overt movement. Specifically, changes in SMR activity resulting from attempted grasping drives the grasp-posture of a stop motion video-based NF paradigm, which forms a strong causal link between intention and action. Our system was also designed to maximize the time participants spent with the grasp-related NF task while maintaining proficient brain control. A short calibration period (<4 min) with no pre-training was sufficient for effective BCI performance. Lastly, task difficulty was adjusted during the session in order to maintain motivation and maximize SMR modulation. We evaluated our system with three participants who had complete hand paralysis due to SCI. We show that in people with complete paralysis, SMR activity can be recorded with MEG during attempted hand movement to drive a real-time NF paradigm and that in some participants (*n* = 2), SMR modulation significantly increased over the course of a session.

## Methods

### Participants and data collection

Three individuals with SCI resulting in complete hand paralysis participated in this study. An occupational therapist evaluated the participants’ injury classification using the ASIA examination (Table 1) [23]. The occupational therapist also confirmed that all participants had no grip strength or active finger flexion or extension using a dynamometer. The subjects all had chronic (i.e. over 1 year since injury) SCI that occurred during adulthood. All subjects were self-reported as right-handed before their injury. Subjects had no previous training with the BCI task. All subjects gave written informed consent and the study was conducted with Institutional Review Board approval from the VA Pittsburgh Healthcare System (VA 02830) and the University of Pittsburgh (PRO09120267).

**Table 1:** Participant demographics and impairment

Subject	Gender	Age	Injury Duration (yrs)	Injury Level	ASIA
S01	Male	31	7	C2	A
S02	Male	26	5	C5	A
S03	Male	27	9	C5	B

MEG was recorded with a 306-channel whole-head system (Elekta Neuromag Vectorview) having 102 sensor-triplets each containing a magnetometer, longitudinal gradiometer, and latitudinal gradiometer. MEG signals were band-pass filtered between 0.1 and 330 Hz and then sampled at 1000 Hz. Standard localization coils were used to track head position relative to the MEG sensors [24]. Head position was recorded after each BCI calibration period (see “BCI calibration task” section below). Based on the recorded head position, each subject’s data was transformed into a standardized sensor coordinate frame to compare between subjects [25].

### BCI system

**Fig. 1:**
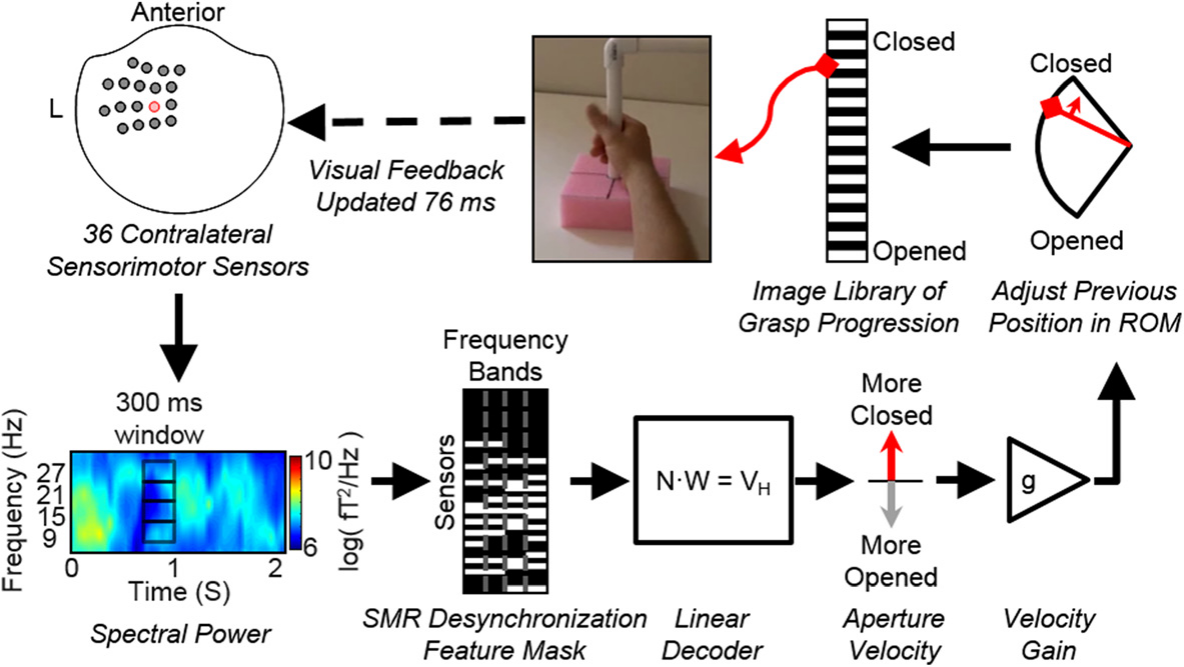
Schematic of the BCI used to translate SMR into proportional control of grasping. Beginning in the upper left, first, the power spectrum of data recorded from 36 sensorimotor MEG sensors (shown on a top-down view of the MEG helmet) are computed using 300 ms sliding windows. A mask is applied to these features to remove any components that did not exhibit desynchronization during calibration. Then a linear decoder applies weights (*W*) to the neural signal (*N*) to compute a hand velocity value (*V*_*H*_). The velocity output from the decoder is scaled (*g*) to ensure movement speeds are appropriate for the task. The previous hand position (an image from the video sequence) is then updated more closed or more opened within the ROM based on the scaled velocity command. The picture representing the desired aperture is chosen from 25 possible images. A progressive change in the images appeared to participants as a grasping movie with a 76 ms refresh rate

Experiments were controlled by a network of computers running a MEG adaptation of our BCI software “Craniux” [26]. Data from 18 sensor locations (i.e. 36 gradiometer signals) over the left sensorimotor cortex (top-left of Fig. 1) were broadcast from the MEG machine in 50 ms bins over TCP/IP using the FieldTrip Buffer [12, 15, 27]. The BCI software sent timestamps via parallel port to the MEG acquisition computer so the full data set (i.e. 306 sensors) could be aligned with the BCI performance information. Spectral decomposition was then performed on data from the 36 sensorimotor sensors using autoregressive functions (25th order Maximum Entropy Method) on a sliding 300 ms bin updated every 50 ms (250 ms of overlap) producing 6 Hz wide frequency bins centered at 9, 15, 21, and 27 Hz (illustrated on the bottom-left of Fig. 1). The window size was chosen in order to balance responsiveness with signal to noise (SNR) ratio.

The goal of the NF training was to promote SMR desynchronization that occurs during overt movement. Therefore, to focus the NF training on neural features that represented SMR desynchronization (i.e. a decrease in power compared to rest), a feature mask was automatically calculated using data collected during calibration to remove any features that showed an increase in SMR power during movement compared to rest (illustrated on the bottom-middle of Fig. 1). That is, any neural feature (four frequency bands × 36 sensors) that did not show desynchronization during calibration was excluded from the neural decoder calculation. In addition, neural features that were unrelated to the calibration task (e.g. noise) were masked if the decoder model fit was below a R^2^ cutoff (either 0.05 or 0.01) for a given feature. After masking, 43 ± 17 % (mean ± std across all calibrations) of the 144 neural features remained for use in the neural decoder. The small percentage of features retained was not surprising considering the large region of interest over the sensorimotor area that was considered.

To proportionally control the hand, linear weights were applied to the spectral power at each time step to move the hand-aperture of the previous time step either more closed or more opened (i.e. the decoder output is driving the incremental displacement of the aperture). This provided an intuitive command strategy that drove the virtual hand more closed during attempted grasp or more opened during rest. Linear regression (minimum norm solution) was used to compute decoder coefficients that related neural features (4 frequency bands on 36 sensors) to the calibration-trial movement states (i.e. +1 for grasp trials, −1 for rest trials) (illustrated on the bottom-middle of Fig. 1). This resulted in a decoder that output a proportional velocity that could move the hand aperture from fully opened to fully closed in 2 s given the SMR levels during the calibration. However, the decoder would output commands with higher speeds as participants learned to better modulate their SMRs. Furthermore, during brain control the grasp speed was scaled linearly to fit the participants’ preference during the first block of the closed-loop task (velocity gain on the bottom-right of Fig. 1). Hand aperture was limited to fully-closed or opened; as is the case with a real hand. The anthropomorphic visualization of movement was created by iterating through sequential images of a real hand grasping with 25 frames between closed and opened (i.e. 4 %-range of motion (ROM) resolution between frames) (illustrated on the top of Fig. 1).

### BCI calibration task

**Fig. 2:**
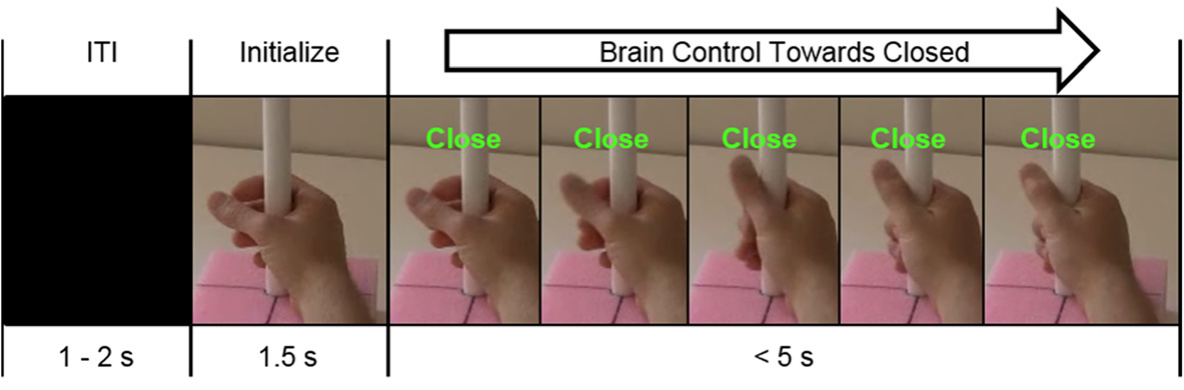
Trial timing. Participants proportionally controlled the hand to an opened or closed target-state during the brain-control phase. A stop motion video of grasping was progressed opened or closed based on brain activity. The full ROM spanned 25 frames of a stop-motion sequence (only 5 shown here). Trials were considered successful if the hand was held within 10 % of the target aperture for the given hold time (minimum of 500 ms)

MEG data were collected during attempted grasp and rest periods in order to determine the weights of the linear decoder that translated SMR power into the proportional control of grasp. During calibration, participants were asked to attempt to grasp, or to rest, along with videos of a hand performing the instructed task. 40 trials of each were collected. We previously determined this number of trials was sufficient for grasp decoding [28]. Each trial consisted of a 1.5 - 2 s inter-trial interval (ITI) of a black screen, followed by 1.5 s of a static hand image at rest, and then a “Close” or “Rest” cue with corresponding video (2 s). Participants were incapable of making overt hand movements, but were asked to attempt the movements during grasp-cues and rest with eyes open at all other times. Subjects were instructed to perform grasps focusing solely on their hand, keeping their arm at rest. In addition, a cylindrical object was used as a goal for grasping (seen in Fig. 2). To minimize eye movements, subjects were instructed to fixate their eyes in the center of the screen throughout each trial.

Calibration of the decoder required less than 4 min of data calibration. Decoder weights were computed using the spectral power in the SMR frequency band (8 – 30 Hz) averaged across each 2 s trial. Subject reaction time was accounted for by removing the first 200 ms after target-cues.

### Brain control task

After calibration, the participants used their SMR activity to control the aperture of the hand in the NF task. As with calibration, they were instructed to attempt to grasp their own paralyzed hand to close the NF hand-display, or to rest their own hand to open the NF hand-display. In each trial, the brain-controlled hand started at 75 % of the ROM away from fully-grasped to encourage more effort and time on the grasping task. Rest trials were used as catch trials to ensure the decoder was not biased towards grasp.

Trial progression was similar to calibration where trials started after a 1.5 - 2 s ITI, which showed a black screen, followed by a 1.5 s hand initialization, which showed a static image of a hand at 75 % open, and a 5 s brain control phase (Fig. 2). The hand initialization phase was used to prevent neural activity from movement-preparation from driving the hand prematurely. Trials were considered unsuccessful if the target was not obtained after the 5 s of brain control or if the hand was held in the opposite target for 3 s. Trials were considered successful if the hand was held within 10 % of the cued closed or opened state for the duration of a hold period. The required hold time was adjusted to increase the difficulty as a way to encourage stronger, sustained SMR modulation. Participants performed 200 trials of closed-loop hand control during a single session. Grasp and rest targets were presented in a pseudorandom block design (20 trials/block) with grasp trials represented more frequently to focus the training on grasp (75 % grasp, 25 % rest trials).

Short breaks (<1 min) occurred every 20 trials during which participants were informed of their performance score and of any increase or decrease in difficulty (i.e. a change in required hold time). Hold time was initially set to 500 ms and was increased by 200 ms if success rates of at least 80 % for grasp (12/15) and 60 % for rest (3/5) were achieved in the 20-trial block. If success rate dropped below 50 %, the hold time was decreased by 200 ms, but limited to a 500 ms minimum. Two participants took extended breaks for pressure relief, which involved moving the MEG chair resulting in a shift in head position. One subject (S02) took an extended break for rest, but did not move within the MEG scanner. The BCI system assumed stationary head position, so the brief calibration process was re-run after breaks involving subject repositioning.

### BCI performance measures

Brain control performance was quantified by calculating the percentage of successful trials. Success rate for only the grasp trials was also reported, as grasp was the focus of the potential intervention. The time required to reach successful grasp-targets was also calculated (i.e. from the end of the initialization phase to the beginning of a successful hold period). False positive rates were assessed to verify that grasp success was not due to a decoder bias. Specifically, Grasp Error Rate was computed as the portion of rest trials that ended with a grasp held for 3 s, indicating that the subject could not prevent grasping. It should be noted that subjects were not instructed to avoid the incorrect target.

A conventional chance level based on the size and number of targets is not an appropriate comparison since the task required a hold period and could end in a time-out. Instead, bootstrapping was used to compute chance levels for each subject based on the decoder outputs that actually occurred. Trial simulations were run for each subject using randomly sampled decoder output values (aperture velocity) that occurred during actual brain control. By sampling from the actual decoder outputs any decoder bias is accounted for. For each time point in the trial simulation, the randomly chosen hand aperture velocity signal was translated into hand movement in the same way that occurred during the actual brain control (i.e. scaled by the speed gain and limited to fully open or fully closed aperture; see Fig. 2). The aperture velocity at each time point was pulled from a grasp or rest trial with equal likelihood. One hundred simulated sessions were generated (i.e. 100 randomizations * 200 trials per subject) and evaluated for success or failure in the same way as actual sessions. Hold times were the same as that defined for each actual trial, and simulated trials were considered failed if the wrong target was held for 3 s. The mean session-wide chance levels from these simulations are reported as well as a breakdown by grasp-only and rest-only trials. One-sided t-tests were used to evaluate if actual success rates fell within the distribution of the simulated sessions for each subject.

### Offline analysis of SMR modulation

SMR modulation during grasp was computed offline to assess if participants were able to alter their brain activity with the NF. Data from all MEG sensors were preprocessed with standard methods available with Neuromag systems. Specifically, bad channels were manually removed before temporal signal-space separation (tSSS) was performed with a 4 s buffer [29]. The head positions for each data set were used to transform sensor data into the default coordinate frame to align sensors across subjects [25]. Data were then resampled to 500 Hz and bandpass filtered between 4 and 120 Hz. The power spectrum of the preprocessed data were computed in a similar manner as they were for BCI, but with higher frequency resolution and across all sensor locations. Specifically, for each trial, the power spectrum was computed from 1 s windows using a 25th order autoregressive model (Burg method) with 1 Hz resolution. Grasp power was calculated from a 1 s window centered at 1.5 s after brain-control initialization to account for reaction time. Baseline rest power was calculated from a 1 s window centered at 1 s before the end of the ITI preceding the trial. SMR modulation was computed as the percent change between grasp and baseline power from 8 to 30 Hz.

The 150 BCI-controlled grasp trials were split into thirds (i.e. first 50 grasps, middle 50, and end 50; deemed “session-segments”) to determine changes in SMR modulation over time. To evaluate the effect NF had on SMR, the maximum modulation magnitude was determined within each gradiometer pair to obtain one modulation value per sensor location in the helmet. It was not necessary that all locations on the left sensorimotor exhibit modulation since spatially focused SMR activity would have been sufficient to drive the neurofeedback. Therefore, the sensor location with the strongest mean SMR modulation across a session was used to evaluate the activity in the left sensorimotor area sensors used for brain control. Repeated measures ANOVA was used to assess the main effect of session-segment, subject, and the interaction of subject and session-segment on SMR modulation. The effect of session-segment on SMR within each subject was assessed with pairwise comparison between the first segment and the subsequent segments (1–2 and 1–3) using Bonferroni multiple comparisons correction.

## Results

### BCI performance

**Fig. 3:**
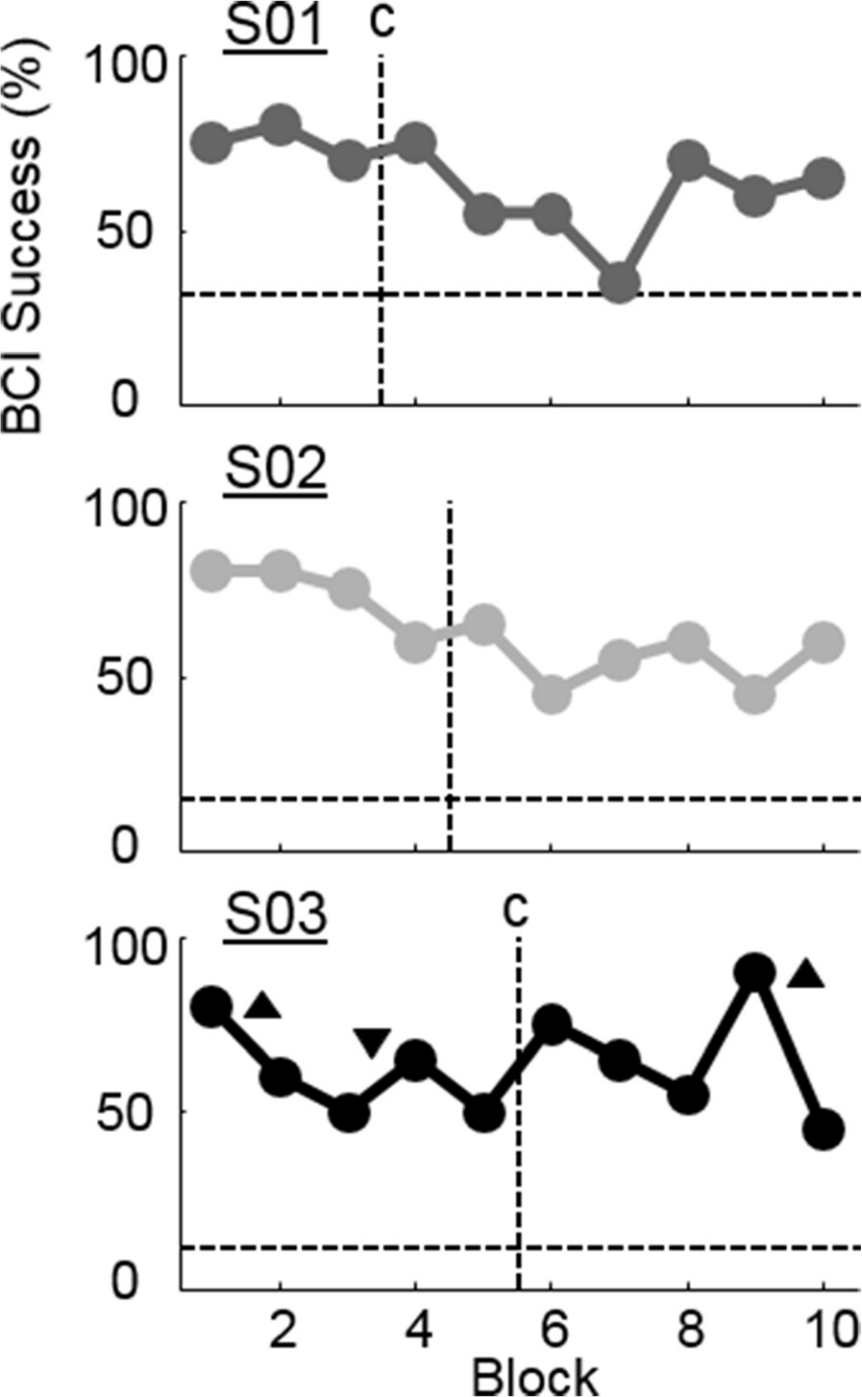
BCI performance across blocks. Mean success rate for each block of 20 trials including 15 grasp and five rest trials. Horizontal dashed lines indicate individual subject chance levels computed with bootstrapping. Vertical dashed lines indicate when breaks happened. A “c” indicates that the decoder was recalibrated during the break. Up arrows indicate that the difficultly was increased by increasing the required hold time from 500 ms to 700 ms. Down arrows indicate the difficulty was decreased to a 500 ms hold time

All subjects were able to control the hand-aperture of the video-based BCI system using SMR activity. Table 2 summarizes the success rate in each session. Grasp success rates varied between 63 and 76 % across subjects while overall success rates varied between 62 and 64 %. Success rate was significantly better than chance for each participant (*p* < 0.001, t-test) with chance levels ranging from 12 to 31 %. Success rate was also significant for each subject when considering grasp-only and rest-only trials (*p* < 0.001, t-test). S01 had a bias toward grasp that was reflected as a higher chance level for grasp trials than other subjects while resting was made more difficult by the bias, which was reflected as a very low chance level for rest trials. To further quantify the effect of a decoder bias towards grasp, Grasp Error Rate was computed as the percent of rest trials that ended incorrectly as a held grasp (Table 2). Table 2 also shows the time taken to reach successful grasps indicating that these trials were performed quickly in 1.96 ± 0.15 s (plus hold time). The time spent on the 200 NF trials was 30, 29, and 31 min (S01, S02, S03 respectively) excluding breaks. Participants took a break for rest and/or pressure relief after 22, 11, and 17 min (S01, S02, and S03 respectively). S01 and S03 repeated the calibration task following the break period to generate a new decoder to account for changes in head position. Though BCI performance improved after breaks for all participants (Fig. 3), the success rate was not significantly higher in the block following a break compared to the block before the break (p = 0.22; paired t-test). The slight improvement in performance could be due to a number of factors including reduced fatigue, renewed motivation, or recalibration (for Subjects S01 and S03). Figure 3 shows the BCI success rate across each block of 20 trials and indicates when recalibrations and breaks occurred.

**Table 2:** BCI performance

Subject	Success (%)	Chance (% ± SEM)	Grasp Success (%)	Grasp Chance (% ± SEM)	Rest Success (%)	Rest Chance (% ± SEM)	Grasp Error Rate (%)	Time to Successful Grasp (s)
S01	64	31 ± .02	76	41 ± .03	28	1 ± .02	10	2.13 ± 1.16
S02	62.5	15 ± .02	66	16 ± .03	52	9 ± .04	2	1.84 ± 1.22
S03	63.5	12 ± 02	63.3	11 ± .02	64	14 ± .04	4	1.90 ± 1.17
Mean ± STD	63.3 ± 0.8	19.3 ± 10.2	68.4 ± 6.7	22.7 ± 16.1	48.0 ± 18.3	8.0 ± 6.6	5.3 ± 4.2	1.96 ± 0.15

### Improvement in SMR modulation

**Fig. 4:**
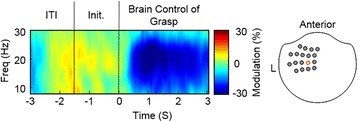
Example signals during brain control of grasp. Average SMR modulation across 150 brain-controlled grasp trials in one sensorimotor sensor for subject S03. This sensor is highlighted in red on a top-down view of the MEG helmet on the right of this figure. At time zero the participant is cued to close the virtual hand by decreasing their SMR, i.e. desynchronization shown as blue. Trials began after an ITI, followed by a hand initialization stage. Modulation is the percent change relative to the SMR activity during the ITI

All participants were able to modulate their SMRs using attempted grasps even though they had complete hand paralysis. As an example, Fig. 4 shows the average spectral activity from a left-sensorimotor sensor during brain control of grasp generated by subject S03. During attempted movement a decrease in SMR activity was observed soon after the target cue was presented (time 0). This would be the expected activation pattern for overt or imagined movement [30, 31].

**Fig. 5:**
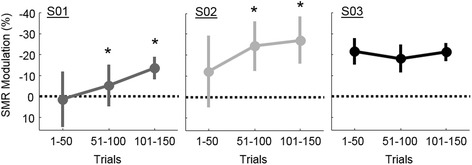
Improvement in SMR modulation across sessions. S01 and S02 show a significant improvement in the ability to modulate SMR compared to their first 50 trials, indicated by the * (*p* < 0.05; corrected for multiple comparisons). Error bars are the standard deviation across trials within each session-segment

**Fig. 6:**
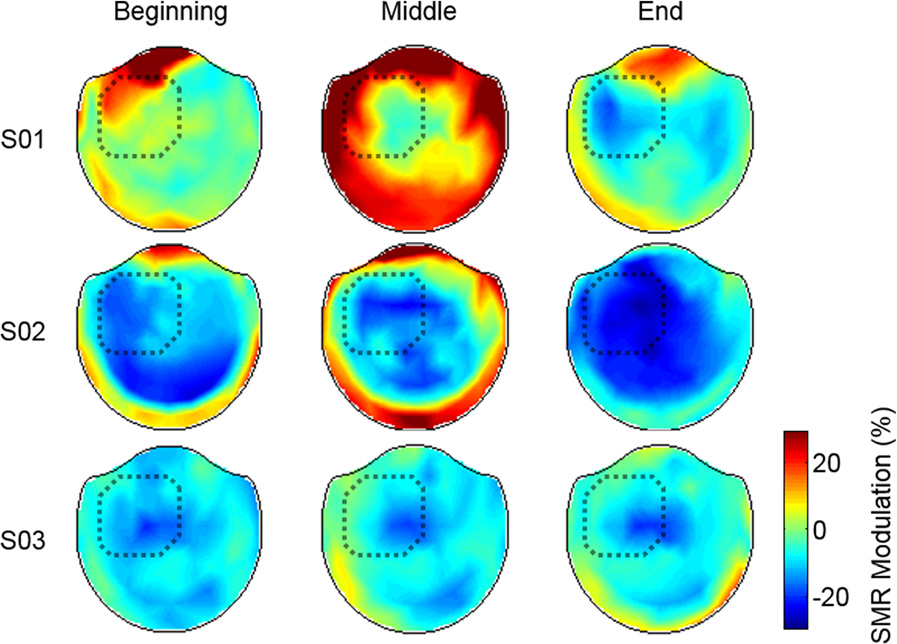
Topography of SMR during the NF session. Changes in SMR modulation across the whole head during the beginning (trials 1–50), middle (trials 50–100), and end (trials 100–150) of NF training. Darker blue indicates stronger desynchronization during BCI grasp control. The location of the sensors used for NF are outlined in dotted lines on a top-down view of the MEG helmet (same as previous figures)

The trends in SMR modulation across the three session-segments are shown in Fig. 5 and 6. When comparing SMR modulation across session-segments with a repeated measures ANOVA, the main effect of session-segment and the interaction of subject and session-segment were significant (*p* < 0.001). The average SMR across subjects increased by 9.9 ± 6.9 pp from the first 50 grasp-trials to the last. Two of the three participants showed a significant increase in their ability to modulate SMRs by 14.9 pp (S01) and 15.0 pp (S02) (*p* < 0.05 pairwise multiple comparisons test with Bonferroni correction). Subject S03 displayed a consistent SMR modulation with no significant differences between session-segments. Figure 6 illustrates the changes in SMR modulation across the whole head. SMR modulation was pronounced in the left-sensorimotor area for all subjects. S01 did not demonstrate a focused desynchronization until the end of the study. The middle session-segment for S01 showed an increase in SMR around the edge of the helmet, which was likely related to neck tension the participant reported. Note that features with an increase in SMR during grasp were masked during calibration of the neural decoder. S02 showed an initial SMR that strengthened as the session progressed while S03 maintained a consistent SMR pattern.

### Discussion

The origins of NF can be traced back many decades to experiments where subjects learned to modulate the activity of their neural oscillations [32] or even single cells recorded from motor cortex [33]. Early clinical applications were focused on neurologic or behavioral conditions such as epilepsy [34], anxiety [35], or attention deficit hyperactivity disorder (ADHD) [36]. The goal of these NF applications was to teach participants to regulate specific brain activity patterns with the goal of returning to a normal baseline. Only recently has NF been applied to motor rehabilitation. We present a novel NF paradigm that has the potential to promote neuroplasticity for motor rehabilitation by utilizing feedback that is driven by neural activation patterns normally expected during movement, in combination with the potential facilitating involvement of the mirror neuron system. Typically, the movement of a cursor or bar is used for continuous feedback in a motor-focused NF paradigm which may limit participant’s ability to embody the NF [10, 11, 14, 37]. Conversely, our calibration and feedback employed anthropomorphic grasping to engage mirror neuron and action observation networks [19, 38], which may have led to stronger SMR desynchronization and embodiment of the NF. We did not explicitly compare anthropomorphic to non-biological feedback, however previous work suggests that using realistic feedback is important for activating the mirror neuron and action observation networks [39–42]. However, future studies are needed to quantify the impact that anthropomorphic feedback has on NF performance and cortical oscillations, especially in a chronically paralyzed population.

Furthermore, the presence of a goal-object (i.e. the grasping bar) likely increased the SMR suppression compared to a non-goal-directed action like simply opening and closing the hand [43]. During brain control, a decrease in SMR-power from attempted grasping of the participant’s own impaired hand drove the stop-motion video towards grasp. This natural and congruent command of grasp was intended to strengthen the causal relationship between intention and feedback. In our study, visual feedback of the realistic hand movement was presented with a 76 ms latency to further maintain a strong causal link between intention and feedback. Delays longer than 200 ms between a person’s movement intention and a device’s reaction is noticeable and can be distracting, leading to degraded task performance [44]. Long delays often seen in BCI-rehabilitation systems may limit their ability to promote plasticity or may make the systems more difficult to control [45, 46]. Due to the limited time for rehabilitation interventions, one of our goals was to minimize the amount of time required for calibration. Quicker system calibration means more time and energy can be devoted to the NF task. Calibration and training time vary widely across other NF studies, ranging from 30 min [37] to multiple training sessions [10, 11] in order to achieve proficient brain control. Our system required less than 4 min of calibration data and no pre-training of participants. Calibration duration was determined based on our previous work which showed that 60 trials (or less than 5 min) of calibration data was needed to decode grasp-intention in able-bodied participants when using multiple frequency bands and sensors from MEG [28]. The addition of our desynchronization feature mask presented here also helped utilize the calibration data by constraining the neural signals to the appropriate SMR modulation as would be expected during movement [30, 31] and by removing noisy features that were inappropriately active during grasp trials. Fast calibration is especially important for MEG-BCI where changes in head position can distort a neural decoder that typically assumes stationary neural activity.

All three participants were able to control grasping and closing of a virtual hand well above chance level, though they were unable to move their own hand due to SCI. This performance was comparable to other SMR-based BCI studies. Similar success rates were found in a MEG-based BCI system for individuals with paralysis due to stroke (between 65 and 90 %) [11]. However, this success rate required multiple sessions to achieve and the NF was a two-choice cursor control task with a 50 % chance level. No improvement in hand function were found after the 13–22 sessions in Buch et al. who enrolled only individuals with complete hand paralysis due to chronic stroke [11]. No changes in hand function were observed after a single session in our study, however, we expect that long term training would be required to achieve functional improvements. Studies with able-bodied participants have shown similar performance quality on a two-choice motor-imagery BCI task demonstrating 74.4 ± 16.5 % accuracy with EEG [37] and 71.74 ± 15.77 % accuracy with MEG [10]. However, a direct comparison is difficult since our study had a hold period and time-out period that made the chance level much less than 50 % (between 12 and 31 %). The grasp error rate calculations indicate that the decoder for subject S01 had a bias towards grasp making it difficult to complete rest trials in spite of good grasp performance. However, S01 did achieve control of both grasp and rest individually that was significantly above chance and had a significant improvement in SMR modulation, which suggests the decoder bias may not have a critical impact on NF.

Two of the three participants demonstrated improved SMR modulation with one session of NF. The changes in SMR occurred locally under the sensorimotor sensors as seen in Fig. 6. The SMR for S03 did not improve significantly, however S03 had stronger SMR modulation during the first session-segment than either S01 (*p* < 0.001, Bonferroni corrected t-test) or S02 (*p* < 0.001); which may have limited the potential for large SMR improvements in only one session.

Though participants demonstrated improved SMR modulation, BCI performance did not improve throughout the session, even when accounting for changes in task difficulty. Though this seems counterintuitive since SMR drove the BCI, a decrease in BCI performance is likely due to decoupling between brain signals and the neural decoder as the session progressed. This change over time is partly due to changes in SMR amplitude and possible reorganization, but also largely due to changes in subject’s head position and comfort. As sessions progressed the participants (who all had had traumatic spinal cord injuries) needed breaks to relax their neck. Fatigue and discomfort can lead to slow changes in head position across a session. This is an important consideration for developing a rehabilitation intervention where patients will have varying impairment. Because the neural decoders in the study assumed a stationary brain within the MEG helmet, the weights likely became sub-optimal in the presence of postural changes. We provided breaks for relaxation and recalibration, but this may not have been enough to mitigate all changes in head-position and alleviate fatigue. In the current study, we recalibrated the system when the participant took a break that would lead to a large change in head position (break timing is indicated in Fig. 3). However, online correction for head movement could potentially eliminate the need to recalibrate and has been demonstrated as feasible [47].

### Conclusions

We present a BCI system that has the potential to promote neuroplasticity for motor rehabilitation. We demonstrated that three of three individuals with complete hand paralysis due to SCI were able to successfully drive the grasping of a virtual hand using SMR activity recorded with MEG. We found that two out of three participants were able to significantly strengthen their SMR activity within one session of NF. This proof of concept study suggests that NF training has the potential to promote neuroplasticity, which could be used for motor rehabilitation. A more extensive study is needed to evaluate if the improvements in SMR are retained and if long term NF training can provide therapeutic benefits to people with different amounts of paralysis. Incorporating design principles that take advantage of biologically-relevant feedback and intuitive command strategies could improve future NF studies.

## Abbreviations

BCI: Brain-Computer Interface
NF: Neurofeedback
SCI: Spinal Cord Injury
MEG: Magnetoencephalography
SMR: Sensorimotor Rhythms

## References

[1] Wilson TW, Fleischer A, Archer D, Hayasaka S, Sawaki L (2011). Oscillatory MEG motor activity reflects therapy-related plasticity in stroke patients. Neurorehabil Neural Repair.

[2] Schaechter JD (2004). Motor rehabilitation and brain plasticity after hemiparetic stroke. Prog Neurobiol.

[3] Tecchio F, Zappasodi F, Tombini M, Oliviero A, Pasqualetti P, Vernieri F (2006). Brain plasticity in recovery from stroke: an MEG assessment. Neuroimage.

[4] Jurkiewicz MT, Mikulis DJ, McIlroy WE, Fehlings MG, Verrier MC (2007). Sensorimotor cortical plasticity during recovery following spinal cord injury: a longitudinal fMRI study. Neurorehabil Neural Repair.

[5] Kokotilo KJ, Eng J, Curt A, Boyd LA (2009). Reorganization and preservation of motor control of the brain in spinal cord injury: a systematic review. J Neurotrauma.

[6] Wang W, Collinger JL, Perez MA, Tyler-Kabara EC, Cohen LG, Birbaumer N (2010). Neural interface technology for rehabilitation: exploiting and promoting neuroplasticity. Phys Med Rehabil Clin N Am.

[7] Grosse-Wentrup M, Mattia D, Oweiss K (2011). Using brain-computer interfaces to induce neural plasticity and restore function. J Neural Eng.

[8] Birbaumer N, Cohen LG (2007). Brain-computer interfaces: communication and restoration of movement in paralysis. J Physiol.

[9] Daly JJ, Wolpaw JR (2008). Brain-computer interfaces in neurological rehabilitation. Lancet Neurol.

[10] Mellinger J, Schalk G, Braun C, Preissl H, Rosenstiel W, Birbaumer N (2007). An MEG-based brain-computer interface (BCI). Neuroimage.

[11] Buch E, Weber C, Cohen LG, Braun C, Dimyan M, Ard T (2008). Think to move: a neuromagnetic brain-computer interface (BCI) system for chronic stroke. Stroke.

[12] Sudre GP, Parkkonen L, Bock E, Baillet S, Wang W, Weber DJ (2011). rtMEG: A Real-Time Software Interface for Magnetoencephalography. Comput Intell Neurosci.

[13] Florin E, Bock E, Baillet S (2014). Targeted reinforcement of neural oscillatory activity with real-time neuroimaging feedback. Neuroimage.

[14] Boe S, Gionfriddo A, Kraeutner S, Tremblay A, Little G, Bardouille T (2014). Laterality of brain activity during motor imagery is modulated by the provision of source level neurofeedback. Neuroimage.

[15] Foldes ST, Wang W, Collinger JL, Li X, Zhang J, Sudre G, et al. Accessing and Processing MEG Signals in Real-Time: Emerging Applications and Enabling Technologies. In: Magnetoencephalography, Edited by Pang EW. ISBN: 978-953-307-255-5, InTech, doi:10.5772/27356. Available from: http://www.intechopen.com/books/magnetoencephalography/accessing-and-processing-meg-signals-in-real-time-emerging-applications-and-enabling-technologies.

[16] Baillet S, Mosher JC, Leahy RM (2001). Electromagnetic brain mapping. IEEE Signal Process Mag.

[17] Ramos-Murguialday A, Broetz D, Rea M, Läer L, Yilmaz O, Brasil FL (2013). Brain-machine interface in chronic stroke rehabilitation: a controlled study. Ann Neurol.

[18] Prasad G, Herman P, Coyle D, McDonough S, Crosbie J (2010). Applying a brain-computer interface to support motor imagery practice in people with stroke for upper limb recovery: a feasibility study. J Neuroeng Rehabil.

[19] Buccino G, Solodkin A, Small SL (2006). Functions of the mirror neuron system: implications for neurorehabilitation. Cogn Behav Neurol.

[20] De Vries S, Mulder T (2007). Motor imagery and stroke rehabilitation: a critical discussion. J Rehabil Med.

[21] Ertelt D, Small S, Solodkin A, Dettmers C, McNamara A, Binkofski F (2007). Action observation has a positive impact on rehabilitation of motor deficits after stroke. Neuroimage.

[22] Iacoboni M, Mazziotta JC (2007). Mirror neuron system: basic findings and clinical applications. Ann Neurol.

[23] Kirshblum SC, Waring W, Biering-Sorensen F, Burns SP, Johansen M, Schmidt-Read M (2011). Reference for the 2011 revision of the international standards for neurological classification of spinal cord injury. J Spinal Cord Med.

[24] Gross J, Baillet S, Barnes GR, Henson RN, Hillebrand A, Jensen O (2013). Good practice for conducting and reporting MEG research. Neuroimage.

[25] Taulu S, Kajola M, Simola J (2004). Suppression of interference and artifacts by the signal space separation method. Brain Topogr.

[26] Wang W, Degenhart AD, Kelly JW, Ashmore RC, Collinger JL, Tyler-Kabara EC (2011). Craniux: A LabVIEW-based modular software framework for brain-machine interface research. Comput Intell Neurosci.

[27] Oostenveld R, Fries P, Maris E, Schoffelen J-M (2011). FieldTrip: Open source software for advanced analysis of MEG, EEG, and invasive electrophysiological data. Comput Intell Neurosci.

[28] Foldes ST, Vinjamuri RR, Wang W, Weber DJ, Collinger JL (2011). Stability of MEG for Real-Time Neurofeedback. Conf Proc IEEE Eng Med Biol Soc.

[29] Taulu S, Hari R (2009). Removal of magnetoencephalographic artifacts with temporal signal-space separation: demonstration with single-trial auditory-evoked responses. Hum Brain Mapp.

[30] Neuper C, Neuper C, Pfurtscheller G, Pfurtscheller G (2001). Evidence for distinct beta reconance frequencies in human EEG related to specific sensorimotor cortical areas. Cinical Neurophysiol.

[31] McFarland DJ, Miner LA, Vaughan TM, Wolpaw JR (2000). Mu and beta rhythm topographies during motor imagery and actual movements. Brain Topogr.

[32] Wyrwicka W, Sterman MB (1968). Instrumental conditioning of sensorimotor cortex EEG spindles in the waking cat. Physiol Behav.

[33] Fetz EE (1969). Operant conditioning of cortical unit activity. Science.

[34] Sterman MB, Friar L (1972). Suppression of seizures in an epileptic following sensorimotor EEG feedback training. Electroencephalogr Clin Neurophysiol.

[35] Hardt J, Kamiya J (1978). Anxiety change through electroencephalographic alpha feedback seen only in high anxiety subjects. Science.

[36] Shouse MN, Lubar JF (1979). Operant conditioning of EEG rhythms and ritalin in the treatment of hyperkinesis. Biofeedback Self Regul.

[37] Blankertz B, Sannelli C, Halder S, Hammer EM, Kübler A, Müller K-R (2010). Neurophysiological predictor of SMR-based BCI performance. Neuroimage.

[38] Grosbras M-H, Beaton S, Eickhoff SB (2012). Brain regions involved in human movement perception: A quantitative voxel-based meta-analysis. Hum Brain Mapp.

[39] Avanzini P, Fabbri-Destro M, Dalla Volta R, Daprati E, Rizzolatti G, Cantalupo G (2012). The dynamics of sensorimotor cortical oscillations during the observation of hand movements: An EEG study. PLoS One.

[40] Collinger JL, Vinjamuri R, Degenhart AD, Weber DJ, Sudre GP, Boninger ML (2014). Motor-related brain activity during action observation: a neural substrate for electrocorticographic brain-computer interfaces after spinal cord injury. Front Integr Neurosci.

[41] Neuper C, Scherer R, Wriessnegger S, Pfurtscheller G (2009). Motor imagery and action observation: modulation of sensorimotor brain rhythms during mental control of a brain-computer interface. Clin Neurophysiol.

[42] Press C, Cook J, Blakemore S-J, Kilner J (2011). Dynamic modulation of human motor activity when observing actions. J Neurosci.

[43] Muthukumaraswamy SD, Johnson BW, McNair N (2004). Mu rhythm modulation during observation of an object-directed grasp. Brain Res Cogn Brain Res.

[44] Welford AT (1968). Fundamentals of Skill.

[45] Lauer RT, Peckham PH, Kilgore KL, Heetderks WJ (2000). Applications of cortical signals to neuroprosthetic control: a critical review. IEEE Trans Rehabil Eng.

[46] Caporale N, Dan Y (2008). Spike timing-dependent plasticity: a Hebbian learning rule. Annu Rev Neurosci.

[47] Little G, Boe S, Bardouille T (2014). Head movement compensation in real-time magnetoencephalographic recordings. MethodsX.

